# Circadian dysregulation induces alterations of visceral sensitivity and the gut microbiota in Light/Dark phase shift mice

**DOI:** 10.3389/fmicb.2022.935919

**Published:** 2022-09-13

**Authors:** Lilin Hu, Gangping Li, Yanyun Shu, Xiaohua Hou, Ling Yang, Yu Jin

**Affiliations:** Division of Gastroenterology, Union Hospital, Tongji Medical College, Huazhong University of Science and Technology, Wuhan, China

**Keywords:** circadian rhythm, light/dark phase shift, visceral hypersensitivity, gut microbiota, intestinal barrier

## Abstract

**Background:**

It is well-established that several features of modern lifestyles, such as shift work, jet lag, and using electronics at night, disturb normal circadian rhythm and increase the risk of suffering from functional gastrointestinal disease. Although substantial evidence demonstrates that shift work is closely correlated with the symptoms of visceral hypersensitivity, few basic studies have revealed the mechanism of visceral hypersensitivity induced by circadian rhythm disturbance, especially light/dark phase shifts. Our study explored the mechanism underlying visceral hypersensitivity caused by light/dark phase shift in mice.

**Methods:**

A 6-h delay light/dark phase shift mice model was constructed. Visceral hypersensitivity was assessed by abdominal withdrawal reflex (AWR) score induced by colorectal distention (CRD) *in vivo* and contraction of colonic muscle strips induced by acetylcholine *ex vivo*. Intestinal permeability was evaluated by transepithelial resistance (TEER) and FD4 permeability. The expression of tight junction proteins was detected by western blotting and immunofluorescence staining. The gut microbiota was examined by 16S rDNA sequencing. Fecal microbiota transplantation (FMT) was performed to confirm the relationship between the light/dark phase shift, gut microbiota, and visceral hypersensitivity.

**Results:**

We found that light/dark phase shift increased visceral sensitivity and disrupted intestinal barrier function, caused low-grade intestinal inflammation. Moreover, we found decreased microbial species richness and diversity and a shift in microbial community with a decreased proportion of *Firmicutes* and an elevated abundance of *Proteobacteria* at the phylum level. Besides, after the light/dark phase shift, the microflora was significantly enriched in biosynthesizing tryptophan, steroid hormone, secondary metabolites, lipids, and lipopolysaccharides. Mice that underwent FMT from the light/dark phase shift mice model exhibited higher visceral hypersensitivity and worse barrier function. Dysbiosis induced by light/dark phase shift can be transmitted to the mice pretreated with antibiotics by FMT not only at the aspect of microbiota composition but also at the level of bacterial function.

**Conclusion:**

Circadian rhythm disturbance induced by the light/dark phase shift produces visceral hypersensitivity similar to the pathophysiology of IBS through modulating the gut microbiota, which may disrupt intestinal barrier function or induce a low-degree gut inflammation.

## Introduction

The mammalian circadian system is composed of the suprachiasmatic nucleus (SCN), a central pacemaker located in the hypothalamus, and several peripheral oscillators distributed throughout the whole organism (Patke et al., 2020). The circadian system consists of interlocking transcription/translation feedback loops that constitute the basis for regulating 24-h behavioral rhythms (Koike et al., 2012). Neurons and astrocytes drive the activity of the central pacemaker in the brain, which coordinates various peripheral oscillators throughout the organism to regulate daily physiological and behavioral changes (Top and Young, 2018). Signals from the external environment can disturb the biorhythm system, and light is the core environmental signal that affects the central circadian clock (Mohawk et al., 2012). Consequently, disrupting the normal 12 h:12 h light/dark cycle leads to circadian disorganization, which may result in serious chronic diseases such as metabolic diseases, neurodegenerative diseases, cancer, and so on (Van Dycke et al., 2015; Stenvers et al., 2019; Steele et al., 2021). In recent years, clinical studies have substantiated that altered circadian rhythms are associated with digestive pathologies. People who work rotating shifts are more likely to suffer from gastrointestinal disorders. In this respect, a meta-analysis revealed that gastrointestinal problems are more common in rotating shift workers than in fixed day shift workers, with an odds ratio (OR) of 1.56 [95% confidence interval (CI): 1.24–1.95] (Chang and Peng, 2021).

An increasing body of evidence from recently published studies demonstrated that rotating shift work is associated with functional gastrointestinal diseases, especially irritable bowel syndrome (IBS) (Nojkov et al., 2010; Kim et al., 2013). Nojkov et al. (2010) found that the incidence of abdominal pain in rotating shift nurses was significantly higher than in day shift nurses (81 vs. 54%, P < 0.0001) and night shift nurses (81 vs. 61%, *P* = 0.003). Moreover, Zhen Lu et al. (2006) demonstrated that nurses on rotating shifts had higher functional bowel disorder (FBD) symptom scores than regular day nurses (0.46 vs. 0.18, *P* = 0.02), rotating shift work was related to sleep disturbances, and somatic pain score was positively related with sleep disturbance score (*r*_s_ = 0.27, *P* = 0.003). In conclusion, rotating work shifts are closely correlated with the symptoms of visceral hypersensitivity, a well-established pathophysiological process of IBS. However, the mechanism of sleep disturbance induced by rotating shift in the pathogenesis of visceral hypersensitivity has been largely understudied, especially in mice models of light/dark phase shift. It seems reasonable to delve into the importance of light/dark cycle disruption as a factor that may participate in the pathophysiology of IBS.

Commensal microbes are involved in intestinal homeostasis, and the dysregulation of host-microbe interactions may lead to the development of local and systemic disorders. Substantial evidence indicates that microbiota dysbiosis plays a key role in the pathogenesis of IBS, inflammatory bowel disease, and metabolism-related disorders (Huang et al., 2018; Vervier et al., 2021). A systematic review revealed that microbiota diversity from IBS patient stool specimens was either decreased or remained unchanged (Pittayanon et al., 2019). Furthermore, several studies demonstrate that circadian disturbance can induce microbiota alterations (Voigt et al., 2014; Cui et al., 2016), but in addition to disrupting the light/dark cycle, they adopted a high-fat, high-sugar diet or radiation treatment. Interestingly, most researchers regard circadian rhythm disorder as a “second hit” and discussed microbiota shift when combined with other interventions. However, little is known currently about the role of gut microbiota in visceral hypersensitivity induced by light/dark phase shift and the alterations in composition and function of intestinal flora and the intestinal physiological function.

In this study, we mainly focused on the circadian rhythm disturbance caused by the light/dark cycle changes and explored the visceral hypersensitivity mechanism induced by light/dark phase shift. A 6-h delay light/dark phase shift mice model was constructed, and intestinal physiological functions, including visceral sensitivity, intestinal barrier function, and intestinal microbiota of this model, were measured. Moreover, fecal microbiota transplantation (FMT) was performed to confirm the relationship among the light/dark phase shift, the gut microbiota, and visceral hypersensitivity.

## Materials and methods

### Animals

#### Light/dark phase shift modeling

In total, 60 7-week-old specific pathogen-free male C57BL/6 mice (Weight: 20 ± 0.5 g) were purchased from the Beijing Vital River Laboratory Animal Technology Co., Ltd (Beijing, China). All animals were raised in the experimental animal center of the Tongji Medical College, Huazhong University of science and technology and fed a standard diet and water *ad libitum* in controlled conditions (temperature of 22^°^C with a 12 h/12 h Light/Dark (8:00–20:00) shift and 60% humidity). All animals were housed adaptively in this environment for 1 week before experiments. All experimental animal procedures were approved by the Animal Experimentation Ethics Committee of Tongji Medical College, Huazhong University of Science and Technology (Ethical approval number: S2659).

**FIGURE 1:**
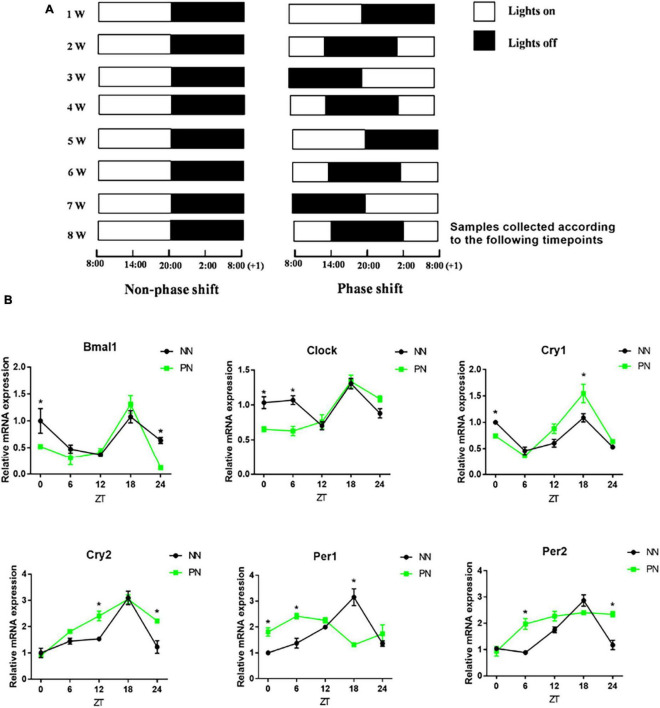
Light/dark phase-shift model. NN and PN mice were kept in a self-made light-controlled feeding box for 8 weeks. **(A)** Lights were turned on and off every week at fixed time intervals in the NN group (Lights on at 8:00 a.m. and off at 20:00). The turning on and off of lights was delayed by 6 h per week in the PN group (Left, NN group; Right, PN group). **(B)** The mRNA expression of clock genes (Bmal1, Clock, Cry1, Cry2, Per1, and Per2) in the colon. Data are expressed as the mean ± SEM. Comparison between two groups performed with a two-tail Student’s *t*-test at each zeitgeber time. *P < 0.05. *N* = 30 per group, *n* = 6 per zeitgeber time. NN, Non-phase shift; PN, Phase shift; ZT, zeitgeber time; Bmal1, aryl hydrocarbon receptor nuclear translocator-like protein 1; Clock, circadian locomotor output cycles kaput; Cry1, cryptochrome 1; Cry2, cryptochrome 2; Per1, period circadian clock 1; Per2, period circadian clock 2.

These 60 mice were randomly divided into Non-phase shift (NN; *n* = 30) and Phase shift (PN; *n* = 30) groups. Each group was divided into five subgroups according to the zeitgeber time expressed as ZT0, ZT6, ZT12, ZT18, and ZT24. There were six mice under each zeitgeber time. We adopted a 6-h phase delay method to induce the light/dark phase-shift model as previously described (Liu et al., 2017). The lights were turned off weekly, and the lighting schedule is described in Figure 1A. The whole modeling cycle was conducted for 8 weeks. Samples were collected at the end of the 8th week according to the five predefined zeitgeber times (ZT0, ZT6, ZT12, ZT18, and ZT24).

#### Fecal microbiota transplantation

We performed FMT to verify the role of microbiota in visceral hypersensitivity caused by light/dark phase shift. Donors were Non-phase shift mice (NN) and Phase shift mice (PN), while recipients were the intestinal decontaminated mice divided into two groups (NF and PF) that were orally gavaged with antibiotics, including vancomycin (100 mg/kg), neomycin sulfate (200 mg/kg), ampicillin (200 mg/kg), and metronidazole (200 mg/kg) for 7 consecutive days (Tang et al., 2021). Fresh feces were collected from NN and PN groups, dissolved in sterile PBS solution, and centrifuged. The supernatant containing fecal microbiota was transplanted to recipient mice by oral gavage three times a week for the whole 8-week modeling period. NF mice received FMT from the NN group and PF mice received FMT from the PN group. There were six mice in each group.

#### Evaluation of visceral hypersensitivity

Colorectal distension (CRD) and the abdominal withdrawal reflex (AWR) score were employed to access the visceral hypersensitivity. The specific process was as follows: The mouse was fixed with the anus exposed, then a self-made catheter with an airbag was slightly inserted into the anus at a depth of 1 cm, the airbag was inserted into the descending colon, and then the catheter was fixed to the mouse tail with adhesive tape. The root of the catheter was connected with a pressure gauge and a 20-ml syringe through a three-way tube adapter. Mice fitted with a catheter were placed in a transparent mouse cage (20 cm × 8 cm × 8 cm) and allowed to adapt for 20 min. A syringe was used to pump gas into the airbag, reaching the predefined pressure (20, 40, 60, and 80 mmHg) and maintained for 20 s. The AWR score under the corresponding pressure was observed and read out by another experimenter, and the operation was repeated two times at an interval of 5 min. The average value of three experiments was taken, and the AWR score was used to reflect the visceral hypersensitivity. At the same time, when the AWR score was 3 points, the corresponding pressure value was referred to as a threshold value of 3 points (also known as the pain threshold). The AWR score was evaluated as previously described (Jin et al., 2018).

#### Contraction of colonic muscle strips induced by acetylcholine

This method was used to assess intestinal visceral sensitivity *ex vivo*, as previously described in our paper (Jin et al., 2018). We used an electrophysiological facility named organ bath to conduct this experiment. During the experiment, the colonic muscle strips were immersed in Kreb’s solution. Muscle strips were suspended in Kreb’s solution until a stable spontaneous contraction curve was observed, then different concentrations of Ach (Sigma, st. Louis, MO, United States, 10^–7^, 10^–6^, 10^–5^, 10^–4^ mol/L) were added to the solution at intervals of 5 min. LabChart software 7.0 (AD Instruments) was used to record the contraction curve of muscle strips. The percentage increase of the area under the curve (AUC) compared to baseline was calculated to reflect the response of colonic muscle strips to Ach in all groups.

#### Histological observation of the colon

Proximal colon tissue of size 1 cm was cut, fixed in 10% paraformaldehyde, and then embedded in paraffin. The paraffin blocks were cut into 5 μm slices and fixed on glass slides, followed by hematoxylin-eosin (HE) staining. Colonic inflammation was observed under an optical microscope (Olympus, Japan). Five visual fields were selected from each slide to determine the degree of inflammation and score. The colonic inflammation score was evaluated as previously described (Feng et al., 2017).

#### Detection of colonic mucosal permeability

We used an electrophysiological device called the Ussing chamber to measure colon mucosal permeability, composed of colon transepithelial resistance (TEER, Ω⋅cm^2^) and FD4 permeability (ng/mL/cm^2^/min). The detection method was described in detail in our previous study (Jin et al., 2018).

#### Western blot

The extraction of total protein from colon tissue was done using RIPA lysate containing protease inhibitor. The concentration of extracted protein was detected by the BCA Kit (Beyotime, Shanghai, China). Equal amounts of protein were added to SDS-PAGE gel for electrophoresis. Then, proteins were transferred to the PVDF membrane after proteins were electrophoretically separated. Then, the PVDF membrane was blocked with 8% skim milk. The protein bands were incubated overnight with Occludin (Invitrogen, Carlsbad, United States), Claudin1 (GeneTex, SAN Antonio, United States), and GAPDH (AntGene, Wuhan, China) antibodies at 4^°^C. Subsequently, all bands were washed three times with TBST and then incubated with the second antibody against horseradish peroxidase at room temperature for 1 h. A highly sensitive ECL chemiluminescence detection kit (Vazyme, Nanjing, China) was used to display protein bands. Protein expression was analyzed by ImageJ 1.8.0 software (NIH, Bethesda, United States).

#### Immunofluorescence of human colon mucosa

Sleep disorders and normal subjects were examined by colonoscopy, and biopsies were taken. The biopsied tissue was embedded in paraffin and sliced. After dewaxing with xylene, the slices were antigen repaired with citric acid buffer. The cell membrane was lysed with 0.3% triton and then blocked with 1% donkey serum at room temperature for 1 h. Each piece of tissue on the slice was incubated in 30 μl of primary antibodies (ZO-1 and occludin), respectively. Then, the slices were put into a wet box and incubated overnight at 4^°^C. After that, slices were washed with PBS three times. Then, each tissue was incubated with 30 μl donkey anti-rabbit green fluorescent antibody 488/FITC at room temperature away from light for 1 h. The nucleus was stained with DAPI for 5 min. Then, the anti-fluorescence quenching agent was dripped and sealed with cover slides. Slices were observed under an inverted fluorescence microscope (Nikon, Tokyo, Japan). The sleep quality of participants was assessed by the Pittsburgh Sleep Quality Index (PSQI) which consisted of 19 self-rated questions (Buysse et al., 1989). These 19 items are grouped into seven component scores, each weighted equally on a 0–3 scale, which has a range of 0–21. Higher scores indicate worse sleep quality; total scores above 11 can be considered as a sleep disorder. This study was approved by the Institutional Ethical Review Committee of Huazhong University of Science and Technology, China (Ethical approval number: H20080308).

#### Real-time fluorescence quantitative PCR

Proximal colon total RNA was extracted using TRIZOL (Takara, Dalian, China) following the manufacturer’s instructions. The concentration and purity of RNA were measured by spectrophotometry. PrimeScript RT Master Mix kit (Takara, Dalian, China) was used to synthesize cDNA. The Roche PCR instrument was used for Real-time fluorescence quantitative PCR. The primer sequences are listed in Supplementary Table 1. The 2^–ΔΔCt^ method was used to analyze the relative expression of target genes normalized to GAPDH.

#### 16S rDNA sequencing

Fecal DNA was extracted using the FastDNA SPIN Kit (Tiangen, Beijing, China) according to the manufacturer’s instructions. The DNA samples underwent 16S rDNA sequencing on a 454 Life Sciences Genome Sequencer FLX platform with titanium (Roche Life Sciences, Basel, Switzerland). The 341F and 806R primers with good specificity and high coverage were used to amplify 16S rDNA regions (V3–V4). The Illumina database building strategy was used to build the 16S database. Each sample was quantified with Qubit 3.0 and pooled into a library to ensure the homogeneity of the sample. Qubit 3.0 and Agilent 2100 were used to perform rigorous quality checks on the library. After the library was qualified, the Illumina high throughput sequencing platform was performed. The α diversity analysis was used to analyze the complexity of microbial community composition in the samples, which can reflect the richness (Chao1, ACE) and diversity (Shannon). Analysis of similarities (ANOSIM) was used to assess the statistical significance of differences in bacterial composition among different groups. The β diversity was adopted to compare and analyze the microbial community structure of different samples, which included Non-Metric Multi-Dimensional Scaling (NMDS), Principal Component Analysis (PCA), and Principal Co-ordinates Analysis (PCoA). The relative abundance of species at phylum, family, and genus levels reflected the community structure among different groups. Linear discriminant analysis (LDA) effect size (LEfSe) was performed to identify species with significant differences in abundance between groups, and results were displayed in an LDA value distribution diagram and cladogram. Finally, PICRUSt software was used to predict the differences in KEGG metabolic pathways among different groups to understand the differences in metabolic pathways of functional genes in microbial communities among groups (Kanehisa et al., 2008). The bioproject accession number of our 16S rDNA sequencing is PRJNA861728.^1^

### Statistical analysis

The data were analyzed by SPSS25.0 software. All results were expressed by mean ± SEM. A comparison between the two groups was performed with a two-tailed Student’s t-test. Pearson correlation analysis was used to analyze the correlation among pain threshold, TEER, FD4 permeability, relative abundance of species at different levels, and abundance of functional genes in different metabolic pathways. A *P*-value < 0.05 was statistically significant. GraphPad Prism 7.0 was used to generate graphs.

## Results

### Light/dark phase-shift model

We used a self-made light-controlled feeding box to model the circadian rhythm disorder induced by light/dark phase shift. The schedule for switching lights on and off in the NN and PN groups is shown in Figure 1A. Then, we examined the expression of colonic clock genes in the NN and PN groups to investigate the effect of light/dark phase shift on colon rhythm. We found that, before ZT12, the expression of Aryl hydrocarbon receptor nuclear translocator-like protein 1 (Bmal1) and Circadian locomotor output cycles kaput (Clock) genes in the PN group decreased relative to the NN group, while the expression of period circadian clock (Pers) genes increased. After ZT6, the expression of the Cryptochrome (Crys) gene in the PN group was increased compared to the NN group. Importantly, the light/dark phase shift did not change the expression pattern of Bmal1, Clock, Cry1, and Cry2 genes throughout the day, while Pers gene expression in the colon was significantly altered (Figure 1B).

### Light/dark phase shift increases visceral sensitivity without microscopic inflammation in mice

**FIGURE 2:**
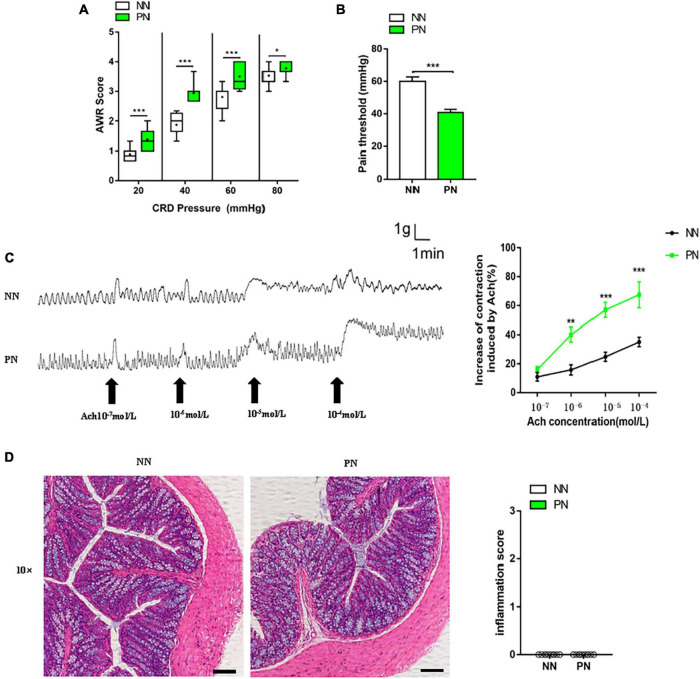
Light/dark phase shift increases visceral sensitivity without microscopic inflammation in mice. **(A)** AWR scores under each CRD pressure. *n* = 12 per group. Data are presented as medians and 25th to 75th interquartile ranges. **(B)** Pain threshold. *n* = 12 per group. **(C)** Contraction curve of colonic muscle strips to Ach and increase in AUC under different Ach concentrations of the NN and PN groups (compared to baseline), *n* = 6 per group. **(D)** HE staining of colon and inflammation score under microscopic observation. Scale bar, 100 μm. *n* = 5 per group. Data are expressed as the mean ± SEM. Comparison between two groups performed with a two-tail Student’s *t*-test. *P < 0.05, **P < 0.01, ***P < 0.001. NN, Non-phase shift; PN, Phase shift; AWR, Abdominal withdrawal reflex; CRD, Colorectal distension; Ach, Acetylcholine; HE, Hematoxylin-eosin staining.

To assess the effect of light/dark phase shift on visceral sensitivity, we first performed CRD and assessed the AWR score. The AWR score under each CRD pressure in the PN group was significantly higher than in the NN group (Figure 2A). Meanwhile, the pain threshold of the PN group was significantly lower than the NN group (Figure 2B). Both indicated that light/dark phase shift increased visceral sensitivity *in vivo*. Then, we used an organ bath to assess the contractility of the colonic strips to Ach *ex vivo*, which can indirectly reflect the visceral sensitivity. A stable spontaneous contraction curve could be observed after the colon muscle strips were immersed in Kreb’s solution for 30 min. Compared to the NN group, faster contraction frequency and higher amplitude were observed in the colon muscle strips of the PN group. Then, after adding different concentrations of Ach at an interval of 5 min, it was found that the reactivity of muscle strips to Ach was stronger in the PN group than in the NN group, especially when the concentration of Ach reached 10^–5^ and 10^–4^ mol/L (Figure 2C). Subsequently, we performed HE staining of proximal colon tissue to assess whether visceral hypersensitivity induced by light/dark phase shift was related to intestinal structural lesions. Microscopic observation showed no inflammatory cell infiltration in the lamina and mucosa, and the inflammatory scores were not comparable between the two groups (Figure 2D). Taken together, the above results suggest that the pathophysiological process of visceral hypersensitivity caused by light/dark phase shift is similar to that of IBS.

### Light/dark phase shift increases intestinal permeability related to the disrupted epithelial barrier

**FIGURE 3:**
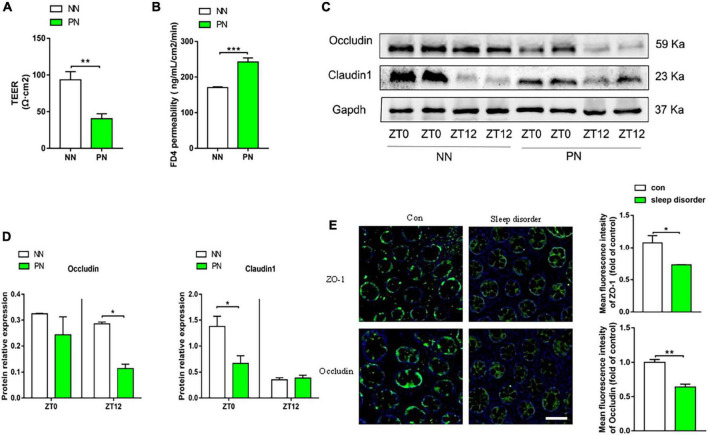
Light/dark phase shift increases intestinal permeability and is related to the disrupted epithelial barrier. **(A)** TEER of colonic mucosa of mice. **(B)** Permeability of mice colonic mucosa to FD4. **(C)** The protein levels of occludin and claudin1 in colon tissue at ZT0 and ZT12 of the NN and PN groups. **(D)** The relative quantitative analysis of protein levels of occludin and claudin1 in mice colon tissue. **(E)** Fluorescence expression of ZO-1 and occludin in the colonic tissue of normal controls and patients with sleep disorders. Scale bar, 100 μm. Data are expressed as the mean ± SEM. Comparison between two groups performed with a two-tailed Student’s *t*-test. *P < 0.05, **P < 0.01, ***P < 0.001. NN, Non-phase shift; PN, Phase shift; TEER, Transepithelial resistance; FD4, FITC-dextran of 4kD; ZT, zeitgeber time.

The pathophysiological process of visceral hypersensitivity usually involves the breakdown of the intestinal barrier, allowing harmful substances to invade the submucosa and increasing intestinal permeability. We first detected transepithelial resistance of the proximal colonic mucosa of these two groups. Results showed that the TEER of the PN group significantly decreased compared to the NN group, which reflected that the integrity of the epithelium was compromised in the PN group (Figure 3A). Subsequently, we calculated the permeability of colonic mucosa to FD4. Moreover, the FD4 permeability of the PN group was elevated compared with the NN group (Figure 3B). These results indicated that light/dark phase shift increased intestinal permeability. We then examined the expression of intestinal epithelial tight junction proteins to explore the molecular mechanism of increased intestinal permeability. The results showed that the protein expression of occludin decreased in the PN group compared to the NN group, especially at ZT12. Similarly, compared with the NN group, the protein expression of claudin1 was reduced in the PN group, especially at ZT0 (Figures 3C,D). We found that light/dark phase shift disrupted intestinal barrier function to increase intestinal permeability. To verify the influence of light/dark phase shift on intestinal barrier function, we detected the fluorescence expression of ZO-1 and occludin in the colonic tissue of normal controls and patients with sleep disorders. Results showed that the fluorescence intensity of ZO-1 and occludin was significantly weakened in sleep disorder patients compared to the control group (Figure 3E).

### Visceral hypersensitivity induced by light/dark phase shift correlates with low-grade intestinal inflammation and epithelial barrier function

During the pathophysiological process of IBS, disruption of the intestinal epithelial barrier increases the passage of harmful microbiota products and antigens across the mucosa, which then result in low-grade inflammation. Accordingly, we detected the mRNA expression of inflammatory cytokines (IL-17α, IL-1β, and IL-6) in colonic tissue of the NN and PN groups. Results showed that the mRNA expression of IL-17α, IL-1β, and IL-6 was significantly upregulated in the PN group than in the NN group, which indicated that the light/dark phase shift also induced a low-grade intestinal inflammation (Supplementary Figure 1). To further evaluate whether visceral hypersensitivity caused by light/dark phase shift was associated with upregulated intestinal inflammation and increased intestinal permeability, we performed a correlation analysis between pain threshold and inflammatory cytokines (IL-17α, IL-1β, and IL-6) as well as TEER and FD4 permeability. Results showed that pain threshold was negatively correlated with the mRNA expression of IL-17α, IL-1β, and IL-6 and FD4 permeability (Supplementary Figures 2A–C,E), while it was positively correlated with TEER (Supplementary Figure 2D). The results indicated visceral hypersensitivity induced by light/dark phase shift correlated with low-grade intestinal inflammation and epithelial barrier dysfunction.

### Light/dark phase shift alters the composition and function of gut microbiota in mice

**FIGURE 4:**
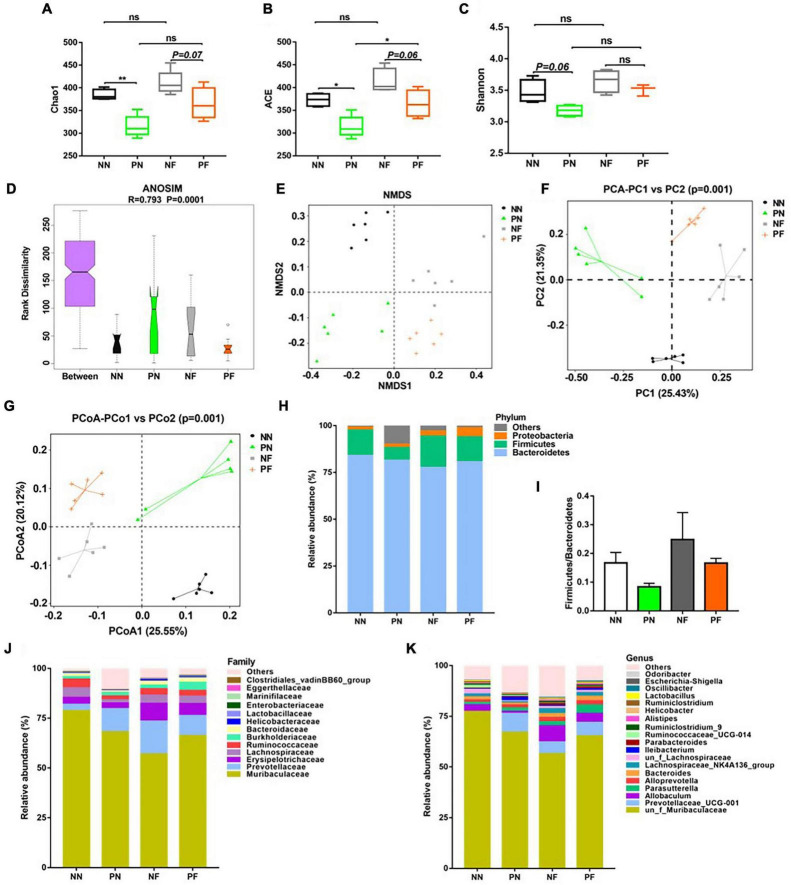
Changes in the structure and composition of gut microbiota caused by light/dark phase shift and FMT. **(A–C)** Comparison of α-diversity indices including **(A)** chao1, **(B)** ACE, and **(C)** Shannon among groups. Data are expressed as mean ± 95% CI. Comparison among groups performed with One-way ANOVA. *P < 0.05; **P < 0.01. **(D)** ANOSIM among groups (*R* > 0, indicating a significant difference between groups). **(E–G)** Comparison of β diversity including **(E)** NMDS, **(F)** PCA analysis, and **(G)** PCoA analysis among groups. Each point represented a sample, and the points with the same color came from the same group. The closer the two points were, the smaller the difference in community structure was. The relative abundance of gut microbiota at the level of phylum **(H)**, family **(J)**, and genus **(K)** among groups. **(I)** The ratio of *Firmicutes*/*Bacteroidetes* among groups. NN vs. PN, NF vs. PF. *n* = 6 per group. NF, mice received FMT from the NN group; PF, mice received FMT from the PN group. NN, Non-phase shift; PN, Phase shift; ANOSIM, Analysis of similarities; NMDS, Non-Metric Multi-Dimensional Scaling; PCA, Principal Component Analysis; PCoA, Principal Co-ordinates Analysis; FMT, fecal microbiota transplantation.

**FIGURE 5:**
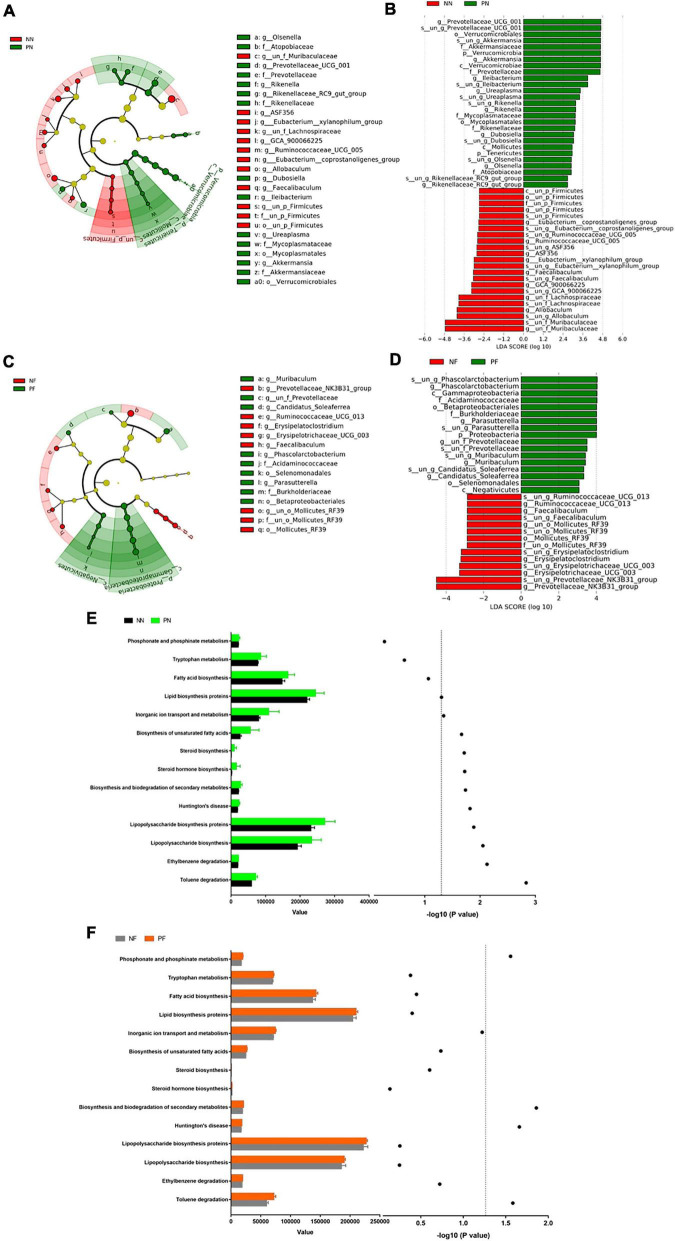
Alternations in the composition and function of gut microbiota caused by light/dark phase shift and FMT. **(A)** Cladogram between NN and PN groups. **(B)** LDA value distribution diagram between NN and PN groups. **(C)** Cladogram between NF group and PF group. **(D)** LDA value distribution diagram between NF group and PF group. **(E)** KEGG metabolic pathway analysis between NN and PN groups. **(F)** KEGG metabolic pathway analysis between NF and PF groups. The left x-axis represents the abundance of functional genes in the metabolic pathway in each group; the right *x*-axis means –log 10(*P*-value). The symbols in panel **(E)** mean the value of –log 10(*P*-value) of the different metabolic pathways between NN and PN groups. The symbols in panel **(F)** mean the value of –log 10(*P*-value) of the different metabolic pathways between NF and PF groups. The dotted line means the value of –log 10(*P*-value) is 1.3. The value of –log 10(*P*-value) >1.3 was considered statistically significant. NN vs. PN, NF vs. PF. *n* = 6 per group. NF, mice received FMT from the NN group; PF, mice received FMT from the PN group. NN, Non-phase shift; PN, Phase shift; LDA, Linear Discriminant Analysis; FMT, fecal microbiota transplantation.

To evaluate the influence of light/dark phase shift on gut microbiota, we performed 16S rDNA sequencing of fecal samples from the NN and PN groups. As shown in Figures 4A–C, the α-diversity indices (including chao1, ACE, and Shannon) of the PN group were significantly decreased than the NN group, which suggested that light/dark phase shift reduce the species richness and diversity of samples. The ANOSIM results revealed that the difference between groups was greater than within groups, and the medians of the NN and PN groups were significantly different (Figure 4D). Next, we analyzed the β diversity of the NN and PN groups to compare the microbial community structure between the two groups. NMDS, PCA, and PCoA showed that gut microbial community structure significantly differed between the NN and PN groups, and microbial composition was significantly influenced by light/dark phase shift (Figures 4E–G). Then, we compared the composition and abundance of gut microbiota in the NN and PN groups at different levels. There were three dominant phyla in both groups: Bacteroidetes, Firmicutes, and Proteobacteria. The PN group had a lower abundance of *Firmicutes* and a higher abundance of *Proteobacteria* than the NN group (Figure 4H). An increasing body of evidence suggests that a reduced *Firmicutes/Bacteroides* ratio is associated with disease susceptibility (Shao et al., 2018; Cui et al., 2021). We calculated the ratio between the NN group and the PN group, which showed that the ratio of F/B was significantly decreased in the PN group than in the NN group, although there was no statistical difference (Figure 4I). At the family level, the PN group had an increased relative abundance of *Burkholderiaceae*, along with a decreased relative abundance of *Erysipelotrichaceae*, *Lachnospiraceae*, and *Ruminococcaceae* (Figure 4J). At the genus level, the relative abundance of genus *Prevotellaceae_UCG-001*, *Parasutterella*, *Alloprevotella*, and *Ileibacterium* was significantly increased in the PN group than in the NN group, while the relative abundance of genus *Allobaculum*, *Lachnospiraceae_NK4A136_group*, *un_f_Lachnospiraceae*, and *Ruminococcaceae_UCG-014* was sharply decreased in the PN group compared with the NN group (Figure 4K). The abundance analysis at different levels found that light/dark phase shift altered the bacterial composition in feces of mice, with an increase in harmful bacteria and a decrease in beneficial bacteria. Subsequently, LEfSe analysis was performed to identify species with significant differences in abundance between these two groups. The cladogram exhibited that *g_un_f_Muribaculaceae*, *g_un_f_Lachnospiraceae*, and *g_Allobaculum* played critical roles and could be regarded as biomarkers of the NN group. At the same time, *g_Prevotellaceae_UCG_001*, *g_Ileibacterium*, and *g_Akkermansia* were the predominant taxa and were considered biomarkers of the PN group (Figure 5A). The LDA value distribution histogram exhibited 17 taxa with significant differences in abundance between the NN group and PN group (| LDA score| > 3.6). Taxa with significant differences at the genus level were consistent with cladogram results between these two groups (Figure 5B). Next, to comprehend the effect of light/dark phase shift on the function of gut microbiota, we performed KEGG metabolic pathway analysis. As shown in Figure 5E, light/dark phase shift had a significant enrichment in the microbial communities which had functional genes in toluene degradation, ethylbenzene degradation, lipopolysaccharide biosynthesis, lipopolysaccharide biosynthesis proteins, Huntington’s disease, biosynthesis and biodegradation of secondary metabolites, steroid hormone biosynthesis, steroid biosynthesis, biosynthesis of unsaturated fatty acids, inorganic ion transport, and metabolism. In conclusion, light/dark phase shift can significantly weaken the abundance of gut microbiota, destroy the structure of intestinal flora, reduce the number of functional bacteria, augment the number of pathogenic bacteria, and produce detrimental metabolites.

### Light/dark phase shift increases visceral hypersensitivity and intestinal permeability by modifying gut microbiota

**FIGURE 6:**
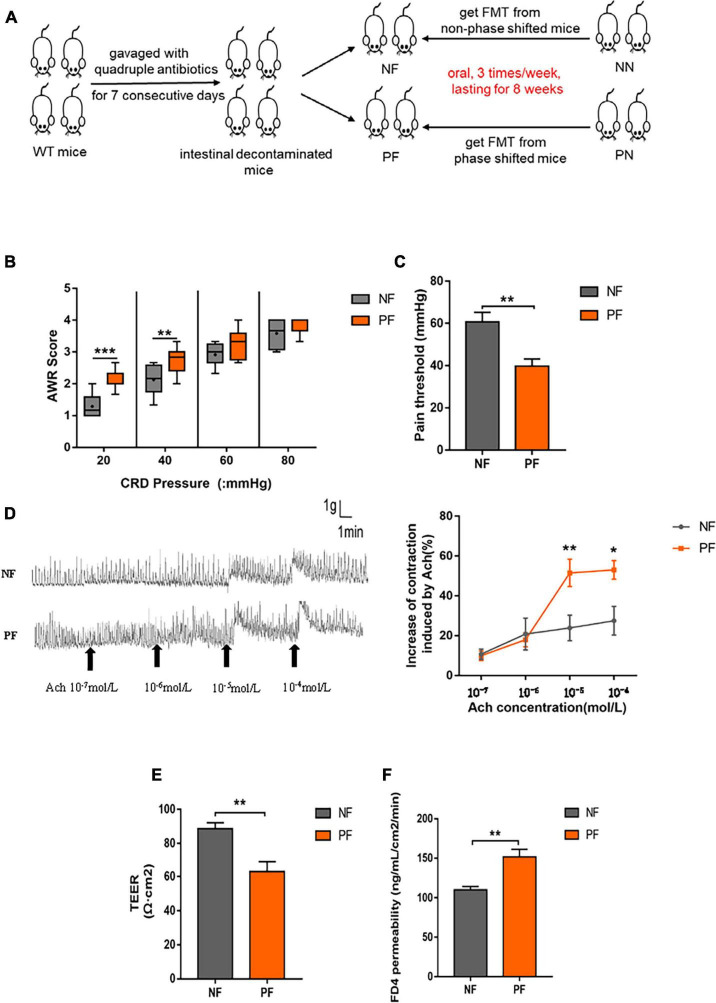
Light/dark phase shift increases visceral hypersensitivity and intestinal permeability by modifying the gut microbiota. **(A)** Schematic of FMT. **(B)** AWR scores under each CRD pressure between the NF and PF groups, *n* = 6 per group. Data are presented as medians and 25th to 75th interquartile ranges. **(C)** The pain threshold of NF and PF groups. *n* = 6 per group. **(D)** Contraction curve of colonic muscle strips to Ach and Proportion of increase in AUC under different Ach concentrations of the NF and PF groups (compared to baseline of each group was calculated). *n* = 6 per group. **(E)** TEER of colonic mucosa. **(F)** Permeability of colonic mucosa to FD4. Data are expressed as the mean ± SEM. Comparison between two groups performed with a two-tailed Student’s *t*-test. *P < 0.05, **P < 0.01, ***P < 0.001. NF, mice received FMT from the NN group; PF, mice received FMT from the PN group; FMT, fecal microbiota transplantation; AWR, Abdominal withdrawal reflex; CRD, Colorectal distension; Ach, Acetylcholine; TEER, Transepithelial resistance; FD4, FITC-dextran of 4kD.

As mentioned above, light/dark phase shift could alter the composition and function of gut microbiota. Then, we carried out FMT to investigate the function of gut microbiota in the intestinal dysfunction caused by light/dark phase shift. The fecal bacteria of the NN and PN groups were transplanted to wild-type C57BL/6 mice that had been pretreated with antibiotics for 1 week by intragastric administration during the modeling cycle of light/dark phase shift (Figure 6A). To exclude the impact of antibiotics treatment on visceral hypersensitivity, we assessed AWR scores and pain threshold of mice pretreated with antibiotics. Results showed that the AWR scores and pain threshold between control mice and antibiotics treated mice had no difference which reflected that the antibiotics pretreatment had no impact on visceral hypersensitivity (Supplementary Figure 3A,B). However, we found that mice of the PF group exhibited higher AWR scores and lower pain threshold than the NF group (Figures 6B,C). The colonic muscle strip response to Ach in *ex vivo* was consistent with the AWR score *in vivo*; the spontaneous contraction curve of colonic muscle strips in the PF group exhibited a larger amplitude and faster frequency than the NF group. Compared with the NF group, the AUC in the PF group was significantly increased when the concentration of Ach reached 10^–5^ mol/L and 10^–4^mol/L (Figure 6D). The results showed that the colonic muscle strips of the PF group had a stronger response to Ach, which further indicated higher visceral sensitivity of the PF group than the NF group. In conclusion, gut microbiota may play a key role in visceral hypersensitivity caused by light/dark phase shift.

Next, to probe the effect of gut microbiota on intestinal permeability, we compared the TEER and FD4 permeability of NF and PF groups. The results revealed that the colonic mucosa in the PF group had lower TEER and higher FD4 permeability compared with the NF group (Figures 6E,F). The results suggest that gut microbiota indeed influenced the destroyed intestinal permeability caused by light/dark phase shift.

### Dysbiosis induced by light/dark phase shift can be transmitted to mice pretreated with antibiotics by fecal microbiota transplantation

The above results showed that light/dark phase shift could increase visceral hypersensitivity and intestinal permeability by modifying the gut microbiota. Next, to verify bacterial colonization after FMT, feces of the NF and PF groups were collected for 16S rDNA sequencing. The chao1, ACE, and Shannon indices were decreased in the PF group compared to the NF group (Figures 4A–C). The ANOSIM results delineated that the medians of the NF and PF groups were significantly different (Figure 4D). NMDS, PCA, and PCoA showed that gut microbial community structure significantly differed between the NF and PF groups (Figures 4E–G). The composition and structure of fecal bacteria after FMT were consistent with the NN and PN groups. The PF group had a higher abundance of *Proteobacteria* and a lower abundance of *Firmicutes* at the phylum level, with a declined ratio of F/B compared with the NF group (Figures 4H,I). Similarly, the composition of bacteria at the family level of the PF group exhibited an increased relative abundance of *Burkholderiaceae*, accompanied by a decreased abundance of *Prevotellaceae, Erysipelotrichaceae*, *Lachnospiraceae*, and *Ruminococcaceae* (Figure 4J). At the same time, the PF group showed a higher abundance of *Prevotellaceae_UCG-001* and *Parassutterella*, with a decreased abundance of *Allobaculum* and *Lachnospiraceae_NK4A136* at the genus level (Figure 4K). To further evaluate whether visceral hypersensitivity caused by light/dark phase shift was associated with some specific bacteria, we performed a correlation analysis between pain threshold and the relative abundance of microbiota at different levels of NF and PF groups. Results showed that pain threshold was negatively correlated with the relative abundance of *p__Proteobacteria*, *f__Burkholderiaceae*, *and g__Parasutterella* (Supplementary Figures 4A,B,D), while it was positively correlated with the relative abundance of *f__Erysipelotrichaceae* and *g__Allobaculum* (Supplementary Figures 4C,E). The results indicated that visceral hypersensitivity induced by light/dark phase shift correlated with microbiota changes. The cladogram demonstrated that *g_Prevotellaceae_NK3B31*, *g_Ruminococcaceae_UCG_013*, and *g_un_o_Mollicutes_RF39* were dominant as biomarkers of the NF group, while *g_Muribaculum* and *g_Parasutterella* were the predominant taxa and can be suggested as biomarkers of the PF group (Figure 5C). The LDA value distribution diagram exhibited 11 taxa with significant differences in abundance between the NF and PF groups (| LDA score| > 4). At the genus level, *g_Prevotellaceae_NK3B31* exhibited significant differences in the NF group. Consistently, the predominant taxa of the PF group were *g_Parasutterella* and *g_Phascolarctobacterium* (Figure 5D). KEGG metabolic pathway analysis (Figure 5F) revealed that functional genes of PF group bacteria were similar to those of the PN group. Next, in order to evaluate whether visceral hypersensitivity caused by light/dark phase shift was associated with these metabolic changes, we performed a correlation analysis between pain threshold and abundance of functional genes in different metabolic pathways of NF and PF groups. Results showed that pain threshold was significantly negatively correlated with the abundance of functional genes in Huntington’s disease, toluene degradation, and phosphonate and phosphinate metabolism (Supplementary Figure 5). The above results indicated that dysbiosis induced by light/dark phase shift could be transmitted to the mice pretreated with antibiotics by FMT, leading to changes in microbiota composition and bacterial function. Moreover, they also reflected that visceral hypersensitivity caused by light/dark phase shift was associated with microbiota changes.

## Discussion

It is widely acknowledged that modern lifestyles, shift work, jet lag, and using electronics at night disturb normal circadian rhythm and increase the risk of cardiovascular diseases, neurodegenerative diseases, metabolic diseases, and cancer (Cui et al., 2016). Disrupted circadian rhythms have been linked to gastrointestinal conditions, including constipation and IBS. Currently, IBS is considered a multifactorial disease associated with visceral hypersensitivity, with an altered relationship between the enteric and central nervous systems. An increasing body of evidence suggests that shift work is closely correlated with the symptoms of visceral hypersensitivity (Nojkov et al., 2010; Kim et al., 2013; Katsifaraki et al., 2019), with few basic studies investigating the mechanism of visceral hypersensitivity induced by circadian rhythm disturbance, especially light/dark phase shift. In the present study, we found that an 8-week 6-h delay light/dark phase shift resulted in visceral hypersensitivity with a higher AWR score and lower pain threshold *in vivo*, faster contraction frequency, and higher amplitude of colon muscle strips *ex vivo*. These findings suggest that circadian rhythm disturbance induced by light/dark phase shift results in visceral hypersensitivity, a vital pathophysiological process in IBS. Subsequently, we revealed that visceral hypersensitivity caused by circadian rhythm disturbance was accompanied by increased intestinal permeability, dysregulated epithelial barrier function, low-grade intestinal inflammation, and changes in composition and function of gut microbiota.

Light is a core environmental signal affecting the central clock and regulating the function of individual internal clock genes. It has been shown that circadian clock gene expression is abnormal after biological rhythm disruption, affecting normal central and peripheral rhythms (Koronowski and Sassone-Corsi, 2021). Overwhelming evidence substantiates that the suprachiasmatic nucleus (SCN) in the hypothalamus regulates and harmonizes the expression of peripheral circadian rhythms, possibly including brain-gut interactions called the brain-gut axis (Bass and Takahashi, 2010; Mohawk et al., 2012; Forsyth et al., 2015), suggesting that environmental signals can affect intestinal rhythm indirectly by regulating the central pacemaker. Our study discovered that light/dark phase shift advanced the trough expression of Bmal1 and Clock genes of colon tissue but did not change the mRNA expression rhythm of the Bmal1, Clock, Cry1, and Cry2 genes throughout the day. While the rhythm of the Pers genes was significantly altered and especially the rhythm of the Per2 gene. These results suggest that circadian rhythm disturbance induced by light/dark phase shift influence the mRNA expression of colonic clock genes and disrupts the normal expression rhythm of the Per2 gene in colonic tissue. However, the role of the Per2 gene in visceral hypersensitivity induced by light/dark phase shift remains unclear, emphasizing the need for further studies.

Circadian rhythm disorganization makes the gut more prone to injury (Forsyth et al., 2015). There are rich literature available suggesting that circadian rhythm disruption can be considered as a “second hit” that increases intestinal permeability when the gut mucosa faces challenges, such as dextran sodium sulfate (DSS) (Preuss et al., 2008; Amara et al., 2019), alcohol (Summa et al., 2013; Swanson et al., 2016), and so on. Indeed, it should be borne in mind that light/dark phase shift alone can risk aggravating intestinal permeability (Summa et al., 2013; Tran et al., 2021). Our study discovered that gut permeability of the PN group (shifted group) was characterized by a decreased TEER and increased FD4 permeability compared with the NN group. Increased intestinal permeability usually indicates a breakdown of gut epithelial barrier structure which was composed of a series of tight junctional (TJ) proteins such as transmembrane proteins—occludin, claudins, and their intracellular domains—zonula occludens 1 (ZO1) protein (Turner, 2009; Suzuki, 2013). Our results showed that the protein expression of occludin decreased in the PN group relative to the NN group, especially at ZT12. Similarly, compared with the NN group, the protein expression of claudin1 was reduced in the PN group, especially at ZT0. The temporal difference in the effect of circadian misalignment on the expression of tight junctional proteins can be attributed to the fact that these markers of intestinal permeability are regulated by circadian rhythms in central and peripheral tissues (Kyoko et al., 2014). Besides, we confirmed that the fluorescence intensity of ZO-1 and occludin was significantly weakened in patients with sleep disorders compared to normal controls. At the same time, circadian perturbance influences gut barrier function and plays a vital role in causing proinflammatory responses. Polidarová et al. (2017) illustrated that a 6-h advance/delay light/dark phase shift (repeated after 2 days) had a significant negative effect on the expression of Rsg16, a gene associated with an anti-inflammatory response, as well as a borderline positive effect on the expression of IL-1α and Stat3 in rat colon. Castanon-Cervantes et al. (2010) also demonstrated that shifted mice exhibited a stronger innate immune response to LPS through activating proinflammatory cytokines in the blood. Consistently, in the present study, we discovered that the mRNA expression of IL-17α, IL-1β, and IL-6 was significantly increased in shifted mice (PN group) compared with the non-shifted group, suggesting that the intestinal immune system was activated in our 6-h delay light/dark phase-shift model. However, histological examination showed no difference in morphology between NN and PN groups. On the one hand, this phenomenon can be attributed to the activation of lamina propria dendritic cells (LPDCs), which are known to play an essential role in regulating the mucosal immune response (Hart et al., 2005; Zhao et al., 2019). Meanwhile, the underlying mechanism of low-grade intestinal inflammation induced by light/dark phase shift warrants further investigation.

Current evidence suggests that barrier dysfunction can trigger immunoregulatory processes, promote mucosal immune activity, and aggravate disease severity and progression (Turner, 2009). Hence, it is important to maintain the integrity of the intestinal barrier under the threat of circadian rhythm disturbances. Indeed, visceral hypersensitivity, intestinal mucosal barrier dysfunction, and intestinal immune activation are all important factors in the mechanism of IBS (Collins and Bercik, 2009). To understand the relationship between visceral hypersensitivity induced by our light/dark phase-shift model, gut barrier function, and gut inflammatory cytokines, we performed a correlation analysis between pain threshold and inflammatory cytokines (IL-17α, IL-1β, and IL-6) as well as TEER and FD4 permeability. Importantly, we found that the pain threshold was negatively correlated with the mRNA expression of IL-17α, IL-1β, and IL-6 and FD4 permeability and positively correlated with TEER. Overall, these results indicated that visceral hypersensitivity induced by light/dark phase shift might correlate with low-grade intestinal inflammation and epithelial barrier dysfunction.

Compelling evidence suggests that gut microbiota plays an important role in maintaining intestinal homeostasis (Maloy and Powrie, 2011; Caricilli et al., 2014; Kayama et al., 2020). Interestingly, the composition and structure of gut microbiota are sensitive and plastic to the host’s gut environment changes, which are linked with a myriad of pathological processes and chronic inflammatory diseases (Voigt et al., 2014; Tang et al., 2017; Fan and Pedersen, 2021). Recent studies revealed that circadian misalignment also generated a negative impact on microbial homeostasis accompanied by an increasing abundance of proinflammatory bacteria as well as a decline of anti-inflammatory bacteria (Voigt et al., 2016a,b), which then perturbed the healthy status of the intestine with a noteworthy possibility to develop inflammation and barrier dysfunction (Khalyfa et al., 2017), consistent with results of the present study. We found that circadian rhythm disorders could contribute to inhibiting microbial species richness and diversity and a shifted microbial community. The results of bacterial relative abundance demonstrated that the phase-shifted mice had a decreased proportion of *Firmicutes* with an increased abundance of *Proteobacteria* at the phylum level compared with non-shifted mice. The gram-positive *Firmicutes* are beneficial bacteria negatively linked with gastrointestinal inflammation except for maintaining intestinal barrier integrity (Cui et al., 2021; Henn et al., 2021). In contrast, gram-negative *Proteobacteria* are defined as a class of main pathogenic bacterium that produce endotoxins (Xiao et al., 2014; Wang et al., 2020) which promote chronic inflammation. Interestingly, at the family level, bacteria *Burkholderiaceae* from *the Proteobacteria* phylum were enriched, while *Erysipelotrichaceae*, *Lachnospiraceae*, and *Ruminococcaceae* from *Firmicutes* phylum were depleted in the shifted mice. Furthermore, our research found that circadian misalignment induced by light/dark phase shift affected the metabolic function of gut microbiota by KEGG metabolic pathway analysis. The enriched microflora of phase-shifted mice increased the function of biosynthesizing tryptophan, steroid hormone, secondary metabolites, lipids, and lipopolysaccharides. It is well-established that tryptophan is catalyzed by tryptophan hydroxylase to produce 5-hydroxytryptophan and then by 5-hydroxytryptophan decarboxylase to produce 5-hydroxytryptophan (5-HT). Moreover, high levels of 5-HT have previously been associated with visceral hypersensitivity in animal experiments and clinical studies (Choi et al., 2008; Keszthelyi et al., 2015; Tong et al., 2020). Besides, abnormal neurotransmitters and endocrine hormones were found to play an essential role in the pathogenesis of visceral hypersensitivity (Hong et al., 2015; Camilleri and Boeckxstaens, 2017). A previous study demonstrated that acute and chronic stress could affect the release of cortisol (human) and corticosterone (rodent) which account for the stress-related gastrointestinal and immune disorders by driving circadian and ultradian bursts of transcriptional activity in the circadian clock gene (Wiley et al., 2016). In conclusion, we concluded that circadian rhythm disturbance induced by light/dark phase shift shaped the composition and function of gut microbiota.

Subsequently, to investigate the function of gut microbiota in the intestinal dysfunction caused by light/dark phase shift, fecal microbiota transplantation was performed. FMT is widely acknowledged to be effective for treating functional gastrointestinal and inflammatory bowel disease (Xu et al., 2021; Zhang et al., 2021). Mounting evidence suggests that FMT can remodel the disease status, such as anxiety and depression-like behaviors (Zheng et al., 2016; Yang et al., 2022). The results of FMT in the present study demonstrated that the mice receiving the fecal bacteria from shifted mice exhibited higher visceral hypersensitivity both *in vivo* and *ex vivo*. Besides, we found that dysbiosis induced by light/dark phase shift can be transmitted to the mice pretreated with antibiotics by FMT, leading to changes in microbiota composition and the level of bacterial function. Importantly, mice in the PF group had a higher abundance of *Proteobacteria* and a lower abundance of *Firmicutes* at the phylum level than the NF group. Similarly, the composition of bacteria at the family level of the PF group exhibited an increased relative abundance of *Burkholderiaceae*, accompanied by decreased abundance of *Prevotellaceae*, *Erysipelotrichaceae*, *Lachnospiraceae*, and *Ruminococcaceae*. Besides, correlation analysis between pain threshold and the relative abundance of microbiota at different levels of NF and PF groups revealed that visceral hypersensitivity induced by light/dark phase shift correlated with microbiota changes. KEGG metabolic pathway analysis revealed that functional genes of PF group bacteria were similar to those of the PN group. And the results of correlation analysis showed that pain threshold was negatively correlated with the abundance of functional genes in biosynthesizing steroid hormone, secondary metabolites, lipids, and lipopolysaccharides, though without a significant difference. Hence, we concluded that light/dark phase shift induces visceral hypersensitivity by modulating mice’s gut microbiota.

Certainly, some limitations of our research need to be considered. First, although the microbiota composition and function of transplanted mice had similarities with those of donors, it remains unclear which kind of bacteria and metabolite play a major role in the process. The bacteria and metabolites negatively correlated with the pain threshold are worthy of further study in future. Second, many other factors also account for visceral hypersensitivities, such as peripheral sensitization of visceral nociceptive receptors, excitation of central visceral sensory neurons, abnormal neurotransmitters, and endocrine hormones apart from the changes in intestinal microbiota. So further studies targeting these factors will endow us with a better understanding of the mechanisms acting at the molecular level and develop new therapeutic targets. In conclusion, our research showed that light/dark phase shift induced visceral hypersensitivity and altered the composition and function of gut microbiota in mice reflected in destroying the structure of intestinal flora, reducing the number of functional bacteria, augmenting the number of pathogenic bacteria, and producing detrimental metabolites. And confirmed that visceral hypersensitivity induced by Light/Dark phase shift was related to the changes of gut microbiota. Hence, life habits respecting circadian rhythms and probiotics are considered to play roles in preventing visceral hypersensitivity or IBS.

## Data availability statement

The datasets presented in this study can be found in online repositories. The names of the repository/repositories and accession number(s) can be found below: https://www.ncbi.nlm.nih.gov/sra/PRJNA861728.

## Ethics statement

The study involving human participants was approved by the Institutional Ethical Review Committee of Huazhong University of Science and Technology, China (Ethical approval number: H20080308). The animal study was approved by the Animal Experimentation Ethics Committee of Tongji Medical College, Huazhong University of Science and Technology (Ethical approval number: S2659).

## Author contributions

LH and YJ: experiments, analysis, and interpretation of data and manuscript drafting. GL and YS: material support. XH and LY: critical revision of the manuscript for important intellectual content. LY and YJ: study supervision, study concept, and design. All authors contributed to the article and approved the submitted version.

## Footnotes

1
https://www.ncbi.nlm.nih.gov/sra/PRJNA861728

## Supplementary material

Supplementary Figure 1 The mRNA expression of inflammatory cytokines [IL-17α **(A)**, IL-1β **(B)**, and IL-6 **(C)**] in colon. *n* = 12 per group. Dates are expressed as the mean ± SEM. Comparison between two groups were performed with a two-tail Student’s *t*-test. **P* < 0.05, ^**^*P* < 0.01. NN, Non-phase shift; PN, Phase shift.

Supplementary Figure 2 Correlation analysis between pain threshold, inflammatory cytokines including IL-17α **(A)**, IL-1β **(B)**, and IL-6 **(C)**,TEER **(D)** and FD4 permeability **(E)**. Person correlation analysis was used. TEER, Transepithelial resistance; FD4, FITC-dextran of 4kD.

Supplementary Figure 3 **(A)** AWR scores under each CRD pressure. *n* = 8 per group. Data are presented as medians and 25th to 75th interquartile ranges. **(B)** Pain threshold. *n* = 8 per group. PBS, PBS pretreated mice; ABX, antibiotics pretreated mice; ns, no significance.

Supplementary Figure 4 Correlation analysis between pain threshold and the relative abundance of *p__Proteobacteria* **(A)**, *f__Burkholderiaceae* **(B)**, *f__Erysipelotrichaceae* **(C)**, *g__Parasutterella* **(D)**, and *g__Allobaculum* **(E)** of NF and PF group. Person correlation analysis was used. NF, mice received FMT from the NN group; PF, mice received FMT from the PN group.

Supplementary Figure 5 Correlation analysis between pain threshold and abundance of functional genes in different metabolic pathways of NF and PF group (according to the results of KEGG metabolic pathway analysis of 16S rDNA sequencing). Person correlation analysis was used. NF, mice received FMT from the NN group; PF, mice received FMT from the PN group.

## Abbreviations

AWR: Abdominal withdrawal reflex
CRD: Colorectal distension
Ach: Acetylcholine
HE: Hematoxylin-eosin staining
TEER: Transepithelial resistance
FD4: FITC-dextran of 4kD
ZT: zeitgeber time
FMT: fecal microbiota transplantation
ANOSIM: Analysis of similarities
NMDS: Non-Metric Multi-Dimensional Scaling
PCA: Principal Component Analysis
PCoA: Principal Co-ordinates Analysis
LDA: Linear Discriminant Analysis
IBS: irritable bowel syndrome
NN: Non-phase shift
PN: Phase shift.
