# Supplementary figures and images for: Circadian dysregulation induces alterations of visceral sensitivity and the gut microbiota in Light/Dark phase shift mice
Source: Front Microbiol. 2022 Sep 13;13:935919. doi: 10.3389/fmicb.2022.935919 (PMC9512646; doi:10.3389/fmicb.2022.935919)

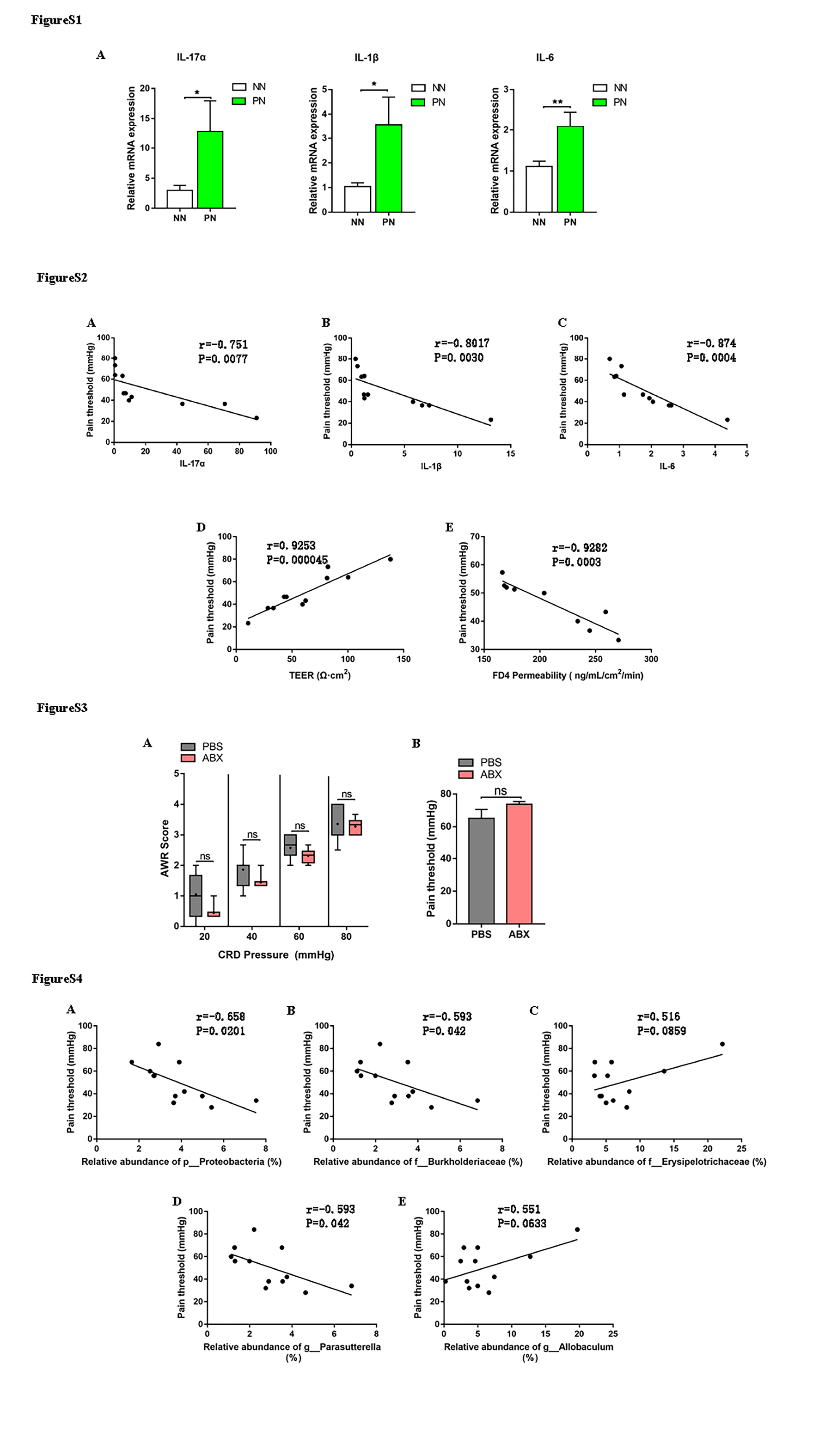

Supplement: Supplementary Figure 1 — The mRNA expression of inflammatory cytokines [IL-17α (A), IL-1β (B), and IL-6 (C)] in colon. n = 12 per group. Dates are expressed as the mean ± SEM. Comparison between two groups were performed with a two-tail Student’s t-test. *P < 0.05, **P < 0.01. NN, Non-phase shift; PN, Phase shift. [file Image_1.TIFF]

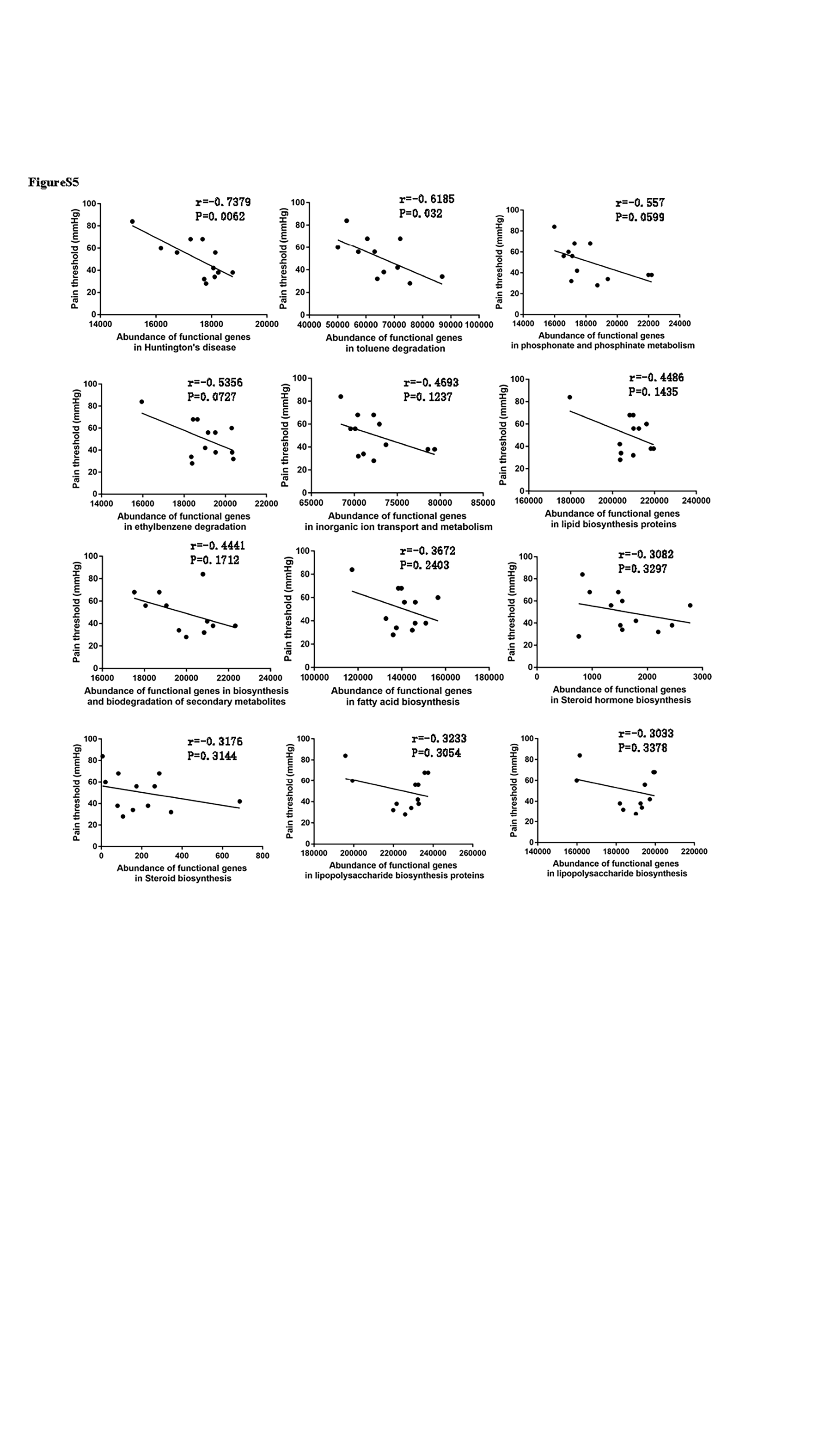

Supplement: Supplementary Figure 5 — Correlation analysis between pain threshold and abundance of functional genes in different metabolic pathways of NF and PF group (according to the results of KEGG metabolic pathway analysis of 16S rDNA sequencing). Person correlation analysis was used. NF, mice received FMT from the NN group; PF, mice received FMT from the PN group. [file Image_2.TIFF]
