# Supplementary material for: Circadian dysregulation induces alterations of visceral sensitivity and the gut microbiota in Light/Dark phase shift mice
Source: Front Microbiol. 2022 Sep 13;13:935919. doi: 10.3389/fmicb.2022.935919 (PMC9512646; doi:10.3389/fmicb.2022.935919)
Supplement: Supplementary file 3 [file Table_1.DOCX]

Table 1. Primer sequences

| Gene | Primer 5’- 3’ |
| --- | --- |
| GAPDH (mouse)  Bmal1 (mouse)  CLOCK (mouse)  Cry1 (mouse)  Cry2 (mouse)  Per1 (mouse)  Per2 (mouse)  IL-17α(mouse)  IL-1β(mouse)  IL-6 (mouse) | Forward：TTCACCACCATGGAGAAGGC  Reverse: GGCATGGACTGTGGTCATGA  Forward：TGACCCTCATGGAAGGTTAGAA  Reverse: GGACATTGCATTGCATGTTGG  Forward：AGAACTTGGCATTGAAGAGTCTC  Reverse: GTCAGACCCAGAATCTTGGCT  Forward：CACTGGTTCCGAAAGGGACTC  Reverse: CACTGGTTCCGAAAGGGACTC  Forward：ACTGGTTCCGCAAAGGACTAC  Reverse: CAGTAGGAACCTCCATCGGTTG  Forward：CGGATTGTCTATATTTCGGAGCA  Reverse: TGGGCAGTCGAGATGGTGTA  Forward：CACACTTGCCTCCGAAATAACTC  Reverse: AGCGCACGGCTGTCTGA  Forward：TTTAACTCCCTTGGCGCAAAA  Reverse: CTTTCCCTCCGCATTGACAC  Forward：CAACCAACAAGTGATATTCTCCATG  Reverse: GATCCACACTCTCCAGCTGCA  Forward：TGATGGATGCTACCAAACTGGA  Reverse: TGTGACTCCAGCTTATCTCTTGG |
